# Hypereosinophilic Syndrome in a Patient With Complex Pulmonary Hypertension and Sjogren’s Syndrome: A Case Report

**DOI:** 10.7759/cureus.78486

**Published:** 2025-02-04

**Authors:** David Wang, Harjeet Singh, Aaron Miller, Christopher D VonTungeln, Linnea Banker

**Affiliations:** 1 Internal Medicine, University of Missouri Healthcare, Columbia, USA; 2 Pulmonary, Critical Care, and Environmental Medicine, University of Missouri Healthcare, Columbia, USA; 3 Pathology, University of Missouri Healthcare, Columbia, USA

**Keywords:** asthma, biologic therapies, hypereosinophilia syndrome, pulmonary hypertension, sjogren’ s syndrome

## Abstract

We describe the case of a 73-year-old man who had been followed up by our clinic for pulmonary hypertension and asthma. He was later hospitalized and found to have significant and persistent eosinophilia compatible with hypereosinophilic syndrome. Various other conditions such as drug reaction with eosinophilia and systemic symptoms (DRESS), malignancy, and eosinophilic granulomatosis with polyangiitis (EGPA) were considered but largely excluded after further investigation. He responded well to steroids and was transitioned to mepolizumab for long-term control.

## Introduction

Hypereosinophilic syndrome (HES) is a rare disorder characterized by significant and persistent blood eosinophilia that results in organ dysfunction [1]. Diagnosis of HES requires that patients have an absolute eosinophil count (AEC) of greater than 1,500 cells/uL on two separate occasions separated by at least one month, evidence of organ damage as a result of eosinophilia, and exclusion of secondary causes that could otherwise explain the organ dysfunction [2]. The exact prevalence of HES is not known, although a study in 2010 showed that it may affect up to 6.3 out of 100,000 individuals. HES can occur at any age, although it most commonly affects young and middle-aged adults [3]. We present a case of a patient with Sjogren’s syndrome, HES complicated by eosinophilic asthma, and complex pulmonary hypertension, who responded to steroids and mepolizumab.

## Case presentation

**Figure 1:**
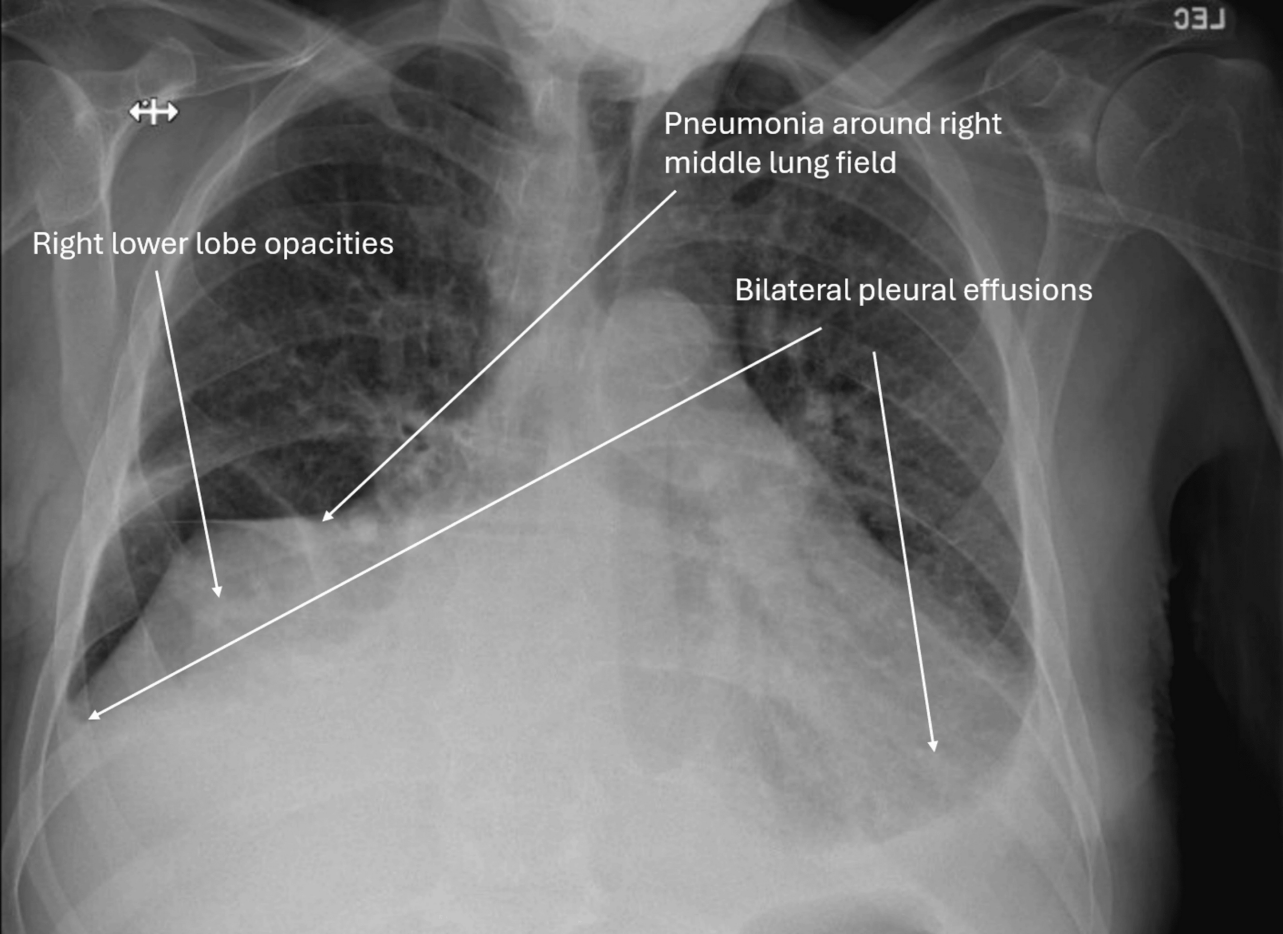
Chest X-ray on admission showing pneumonia around the right middle lung field, new opacities in the right lower lobe, mild interstitial edema, and bilateral pleural effusions.

**Figure 2:**
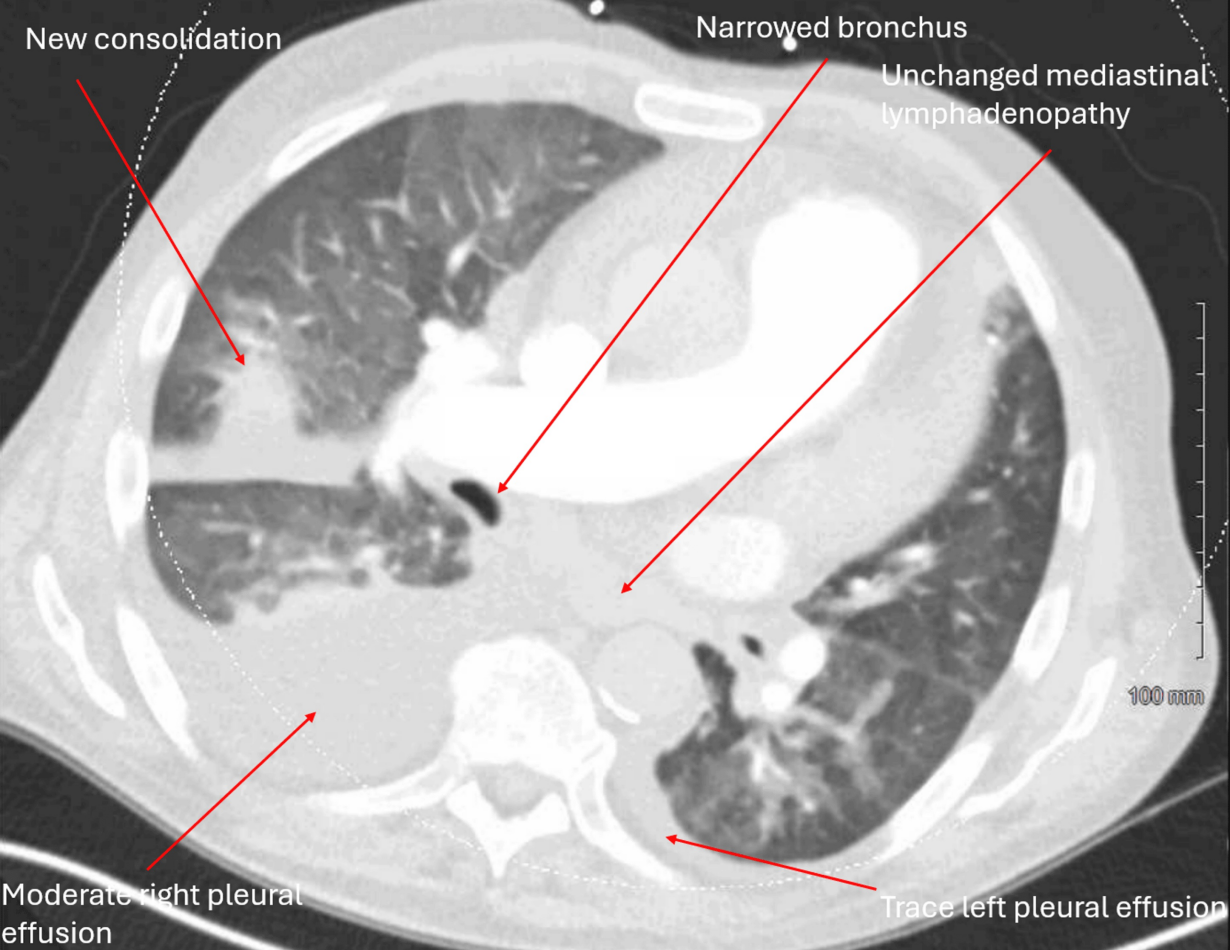
CT pulmonary angiogram with IV contrast on 10/24/22 showing no evidence of PE, a new consolidation in the right lower lobe, improving right middle lobe mass-like consolidation with persistent narrowing of the right middle lobe bronchus, increasing moderate right pleural effusion, small left pleural effusion, and unchanged mediastinal lymphadenopathy.

**Figure 3:**
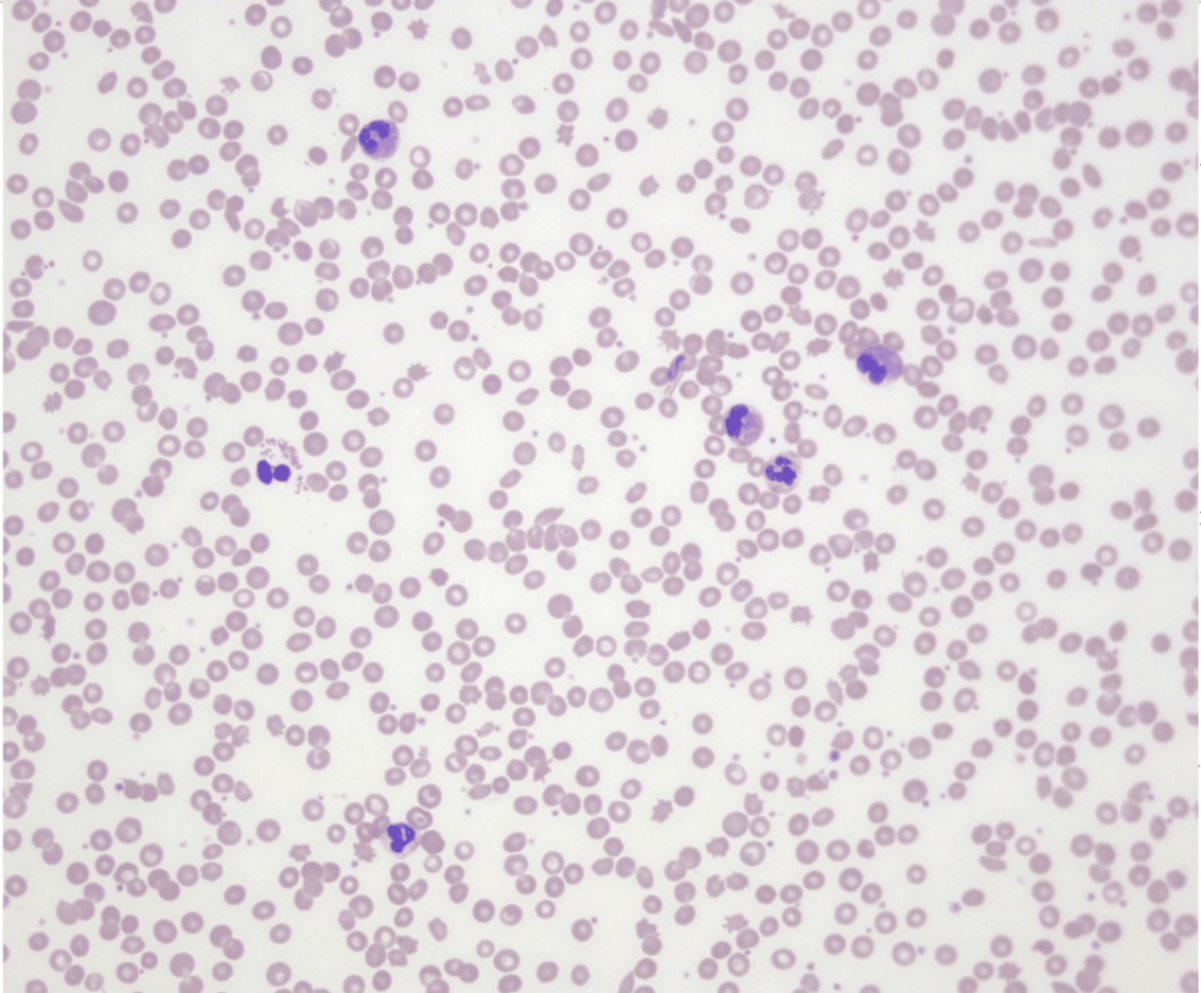
Peripheral smear showing eosinophilia and normocytic, normochromic anemia

**Figure 4:**
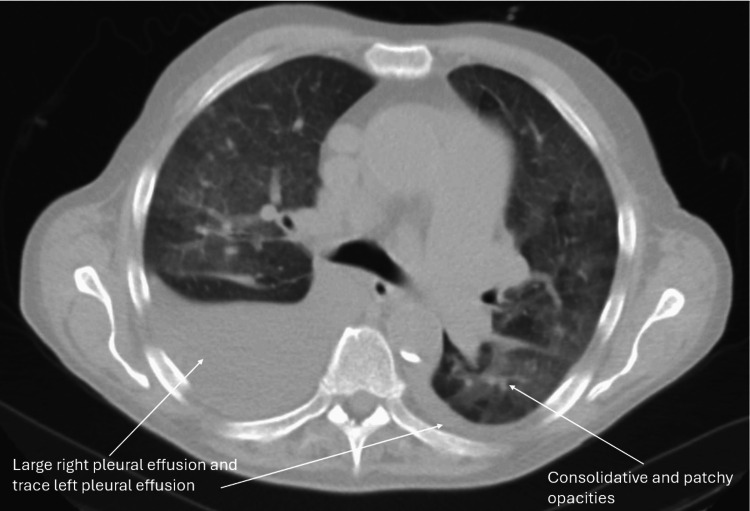
PET scan showed findings consistent with multifocal pneumonia, moderate-to-large right pleural effusion, trace left pleural effusion, and mediastinal and right hilar lymphadenopathy that was likely reactive to infection or inflammation. PET, positron emission tomography

A 73-year-old male with a history of pulmonary hypertension, heart failure with rec, relapsing polychondritis, asthma, atrial fibrillation and Sjogren's syndrome presented to the hospital for worsening dyspnea. The patient experienced chronic dyspnea at baseline but noticed that it had gotten significantly worse around two weeks prior to visiting the hospital. He normally wears 2-3 liters of supplemental oxygen but required 6 liters upon admission. Physical examination was notable for mild jugular venous distension, regular rate and rhythm of heart, normal inspiratory effort, crackles in the right middle/lower lobes, and 2+ pitting edema of the lower extremities. Chest X-ray (Figure 1) showed persistent right middle lobe pneumonia, new opacities in the right lower lobe, mild interstitial edema, and bilateral pleural effusions. Computed tomography (CT) pulmonary angiogram with IV contrast (Figure 2) showed a new consolidation in the right lower lobe, improving right middle lobe mass-like consolidation with persistent narrowing of the right middle lobe bronchus, increasing moderate right pleural effusion, small left pleural effusion, and unchanged mediastinal lymphadenopathy (Figure 1). Labs upon admission were notable for a white blood cell (WBC) count of 11.79K/uL, low hemoglobin of 8.3 g/dL, low sodium of 126 mmol/L, low potassium of 3.2 mmol/L, glucose of 122 mg/dL, creatinine of 0.88 mg/dL, slightly elevated alkaline phosphatase (ALP) of 137 units/L, normal aspartate aminotransferase (AST) of 24 units/L, normal alanine aminotransferase (ALT) of 22 units/L, and low iron saturation of 18% (Table 1). Absolute eosinophil count (AEC) was 5,660 and first noted to be above 1,500 around this time. Percent eosinophils was 48% on admission. Home medications the patient was taking at the time of admission included albuterol, allopurinol, apixaban, fluticasone furoate inhalation powder, atorvastatin, carvedilol, fluticasone nasal spray, furosemide, pilocarpine, potassium chloride, sildenafil, Uptravi, and vitamin B12. He did not have noticeable skin lesions, rash, or neuropathy. Peripheral blood smear (Figure 3) showed an increased number of eosinophils with normal morphology, normocytic normochromic anemia, and mild lymphopenia (Figure 2). Antineutrophil cytoplasmic antibodies (ANCA), anti-myeloperoxidase (MPO), and anti-proteinase 3 (PR3) were negative. The total IgE level was elevated to 445 kilounits/L. Antinuclear antibodies (ANA) were found to be positive to 1:1,280 earlier that year. A positron emission tomography (PET) scan was obtained to rule out underlying malignancy, which showed findings consistent with multifocal pneumonia, moderate-to-large right pleural effusion, trace left pleural effusion, and mediastinal and right hilar lymphadenopathy that was likely reactive to infection or inflammation (Figure 4). Sputum cytology showed acute inflammation with necro-inflammatory debris, increased eosinophils, and no malignant cells. Sputum culture was positive for *Staphylococcus aureus* and *Stenotrophomonas maltophilia*. Vancomycin was administered. The patient underwent a right-sided thoracentesis a few days after admission, and 1,600 milliliters of serosanguineous fluid was removed. The effusion was found to be transudative in etiology with 69% percent eosinophils. Pleural fluid cytology showed mesothelial cells with reactive changes, markedly increased eosinophils, and no malignant cells. Pleural fluid cultures showed no growth. Flow cytometry was performed several days after admission, which showed 92.9% CD3 T cells (elevated), 58.9% CD4 T helper cells, 31.2% CD8 T suppressor cells, and a CD4/CD8 ratio of 1.88%. Electrolytes were monitored while he was hospitalized. Hypokalemia and hyponatremia largely resolved by the time of his discharge. Steroids were administered for potential treatment of acute versus chronic eosinophilic pneumonia and eosinophilic granulomatosis with polyangiitis (EGPA). Percent eosinophils decreased to normal shortly after steroids were started, and he was discharged home on a prednisone taper. He felt significantly better after starting steroids.

**Table 1:** Lab values upon admission *Notable values ALP, alkaline phosphatase; ALT, alanine aminotransferase; AST, aspartate aminotransferase; FANA, fluorescent antinuclear antibody; WBC, white blood cells

Variables	Value	Reference range
Sodium, nmol/L	126*	136-145
Potassium, nmol/L	3.2*	3.5-5.1
Glucose, mg/dL	122	70-139
Creatinine, mg/dL	0.88	0.7-1.2
Alkaline phosphatase, units/L	137*	40-129
Aspartate aminotransferase, units/L	24	<40
Alanine aminotransferase, units/L	22	10-50
WBC, K/uL	11.79K	3.5-10.5
Hemoglobin, g/dL	8.3*	13.5-17.5
Iron saturation, %	18%*	20-55
Absolute eosinophil count, cells/uL	5,660*	50-500
Percent eosinophils, %	48%*	NA
Total IgE, kilounits/L	445*	0-127
FANA	1:1,280*	<1:80

The patient was admitted to the hospital around three months later for reasons unrelated to HES. AEC was as high as 1,540 during this admission. ANCA, anti-MPO, and anti-proteinase 3 (PR3) tests were unremarkable at this time. By discharge, his eosinophil percentage decreased to 10%. The patient’s eosinophil count remained relatively stable until around two months later when he was hospitalized for dehydration and hypokalemia. AEC was as high as 1,190. Prednisone was started on hospital day 4 and AEC decreased to 140 on the day of discharge. He was later weaned off prednisone and was started on mepolizumab. Since then, his AEC has been relatively stable.

## Discussion

Patients with HES may present with various symptoms including cough, dyspnea, fatigue, weakness, rash, and diarrhea, although there is significant heterogeneity in clinical presentation. HES may be asymptomatic in some individuals and progress to leukemia in others [4]. Ground glass, patchy infiltrates may be seen on CT or chest X-ray. Other clinical manifestations include pulmonary emboli, pulmonary nodules, cardiac disease, pleural effusions, and intrathoracic lymphadenopathy [5]. While mild elevations in eosinophils can be seen in Sjögren's syndrome, significant eosinophilia resulting in organ dysfunction is extremely rare in Sjögren's syndrome [6]. Some of the common cardiac manifestations of HES include arrhythmias, heart failure, cardiac thrombi, and myocardial ischemia [7]. Our patient had heart failure with recovered ejection fraction and atrial fibrillation. Our patient did not have an endomyocardial biopsy, but HES may have played a role in his atrial fibrillation and heart failure.

Certain clinical and lab findings are associated with a poor prognosis in HES, such as cardiac involvement, elevated levels of vitamin B12, leukocytosis greater than 100K/cm3, anemia, and thrombocytopenia [3]. However, specific biomarkers of disease progression have not yet been identified [8]. Our patient also has a history of asthma, but his marked degree of eosinophil elevation was more consistent with HES than eosinophilic asthma. The patient’s absolute eosinophil count was also greater than 1,000 during times when he did not have symptoms of an asthma exacerbation. EGPA was unlikely as the patient's ANCA was negative. A transverse colon biopsy and salivary gland biopsy in the past did not show any evidence of eosinophilic infiltration or necrotizing vasculitis.

Management of HES depends on the degree of lab abnormalities present and severity of a patient’s symptoms [8,9]. Patients with symptoms of hyperleukocytosis involving the pulmonary and neurologic systems as well as an AEC above 100K cells/uL qualify as having severe HES and should be treated with high-dose steroids. For patients who are asymptomatic and have an AEC of less than 1,500, close monitoring is sufficient [8,9]. Systemic steroids are the main line of therapy for most variants of HES. Other potential treatments include hydroxyurea and biologics, such as mepolizumab and benralizumab, for patients unresponsive to steroids or those who require high doses of steroids [3,8]. Mepolizumab targets and blocks the cytokine, interleukin-5 (IL-5). This reduces the number of eosinophils and associated inflammation as IL-5 is essential for eosinophil survival and recruitment [10]. Benralizumab is a monoclonal antibody that targets the IL-5 receptor alpha subunit and destroys eosinophils through antibody-mediated recruitment of natural killer cells [11].

## Conclusions

HES, while rare, should be considered in patients presenting with significantly elevated eosinophil levels and signs of organ dysfunction. Recognition of this condition can help facilitate timely treatment. This case also illustrates the importance of follow-up with patients and completing an extensive workup of lab abnormalities.
